# Identification and characterization of *TOR* in *Macrobrachium rosenbergii* and its role in muscle protein and lipid production

**DOI:** 10.1038/s41598-023-50300-3

**Published:** 2024-01-24

**Authors:** Xilin Dai, Xuenan Li, Danhui Yin, Xin Chen, Linwei Wang, Luyao Pang, Yuanshuai Fu

**Affiliations:** 1 Key Laboratory of Freshwater Aquatic Genetic Resources, Ministry of Agriculture and Rural Affairs, Shanghai Ocean University, Shanghai, 201306 China; 2 Shanghai Collaborative Innovation for Aquatic Animal Genetics and Breeding, Shanghai, 201306 China; 3 National Experimental Teaching Demonstration Centre for Aquatic Sciences, Shanghai Ocean University, Shanghai, 201306 China

**Keywords:** Cell biology, Molecular biology, Physiology

## Abstract

The recent scarcity of fishmeal and other resources means that studies on the intrinsic mechanisms of nutrients in the growth and development of aquatic animals at the molecular level have received widespread attention. The target of rapamycin (TOR) pathway has been reported to receive signals from nutrients and environmental stresses, and regulates cellular anabolism and catabolism to achieve precise regulation of cell growth and physiological activities. In this study, we cloned and characterized the full-length cDNA sequence of the *TOR* gene of *Macrobrachium rosenbergii* (*MrTOR*). *MrTOR* was expressed in all tissues, with higher expression in heart and muscle tissues. In situ hybridization also indicated that *MrTOR* was expressed in muscle, mainly around the nucleus. RNA interference decreased the expression levels of *MrTOR* and downstream protein synthesis-related genes (*S6K*, *eIF4E*, and *eIF4B*) (*P* < 0.05) and the expression and enzyme activity of the lipid synthesis-related enzyme, fatty acid synthase (FAS), and increased enzyme activity of the lipolysis-related enzyme, lipase (LPS). In addition, amino acid injection significantly increased the transcript levels of *MrTOR* and downstream related genes (*S6K*, *eIF4E*, *eIF4B*, and *FAS*), as well as triglyceride and total cholesterol tissue levels and FAS activity. Starvation significantly increased transcript levels and enzyme activities of adenylate-activated protein kinase and LPS and decreased transcript levels and enzyme activities of FAS, as well as transcript levels of *MrTOR* and its downstream genes (*P* < 0.05), whereas amino acid injection alleviated the starvation-induced decreases in transcript levels of these genes. These results suggested that arginine and leucine activated the TOR signaling pathway, promoted protein and lipid syntheses, and alleviated the pathway changes induced by starvation.

## Introduction

The increasing scarcity of fishmeal and fish oil resources means that research into precision nutritional feeding for aquatic animals has become an urgent issue^1^. The development of molecular nutritional research provides us with a new perspective, and in-depth research into aquatic animal nutrition has also led people to recognize the close interaction between nutrients and gene expression at the molecular level. Elucidating the regulatory mechanisms of nutrients and nutritional regulatory factors in terms of the physiological functions of animals will effectively and economically promote their growth and development, improve their resistance to diseases, and maximize the realization of their genetic potential^2,3^.

Target of rapamycin (TOR) protein was originally discovered in budding yeast during the study of resistance mutations to the fungal toxin and immunosuppressant, rapamycin. TOR responds to changes in nutritional conditions and regulates growth in yeast^4^. The TOR protein is a large, multi-domain serine/threonine kinase belonging to the phosphatidylinositol-3-kinase-related kinase family, containing a 16–20 amino acid N-terminal HEAT structural domain, a centrally located focal adhesion targeting (FAT) structural domain, an FKBP12-rapmaycin-binding domain, a C-terminal kinase structural domain, and a focal adhesion targeting domain of C-terminal (FATC) domain^5^. TOR proteins are conserved and widely present in vertebrates and invertebrates^6^. TOR exists in the cell in the form of two complexes, TORC1 and TORC2, which sense different external signals and play different functions in the cell by phosphorylating different substrates. TORC1 is mainly involved in the regulation of growth^7^, whereas TORC2 mainly participates in construction of the actin cytoskeleton^8^. A variety of factors upstream of the TOR protein, such as rapamycin, growth factors, environmental stress, and nutrition, can regulate the function of TORC1^9^, while many proteins involved in protein synthesis, lipid metabolism, and energy metabolism downstream of TORC1 are affected by the TOR signaling pathway to regulate cell growth and proliferation^10^. TOR thus acts as a general switch of anabolism and plays a central role in the nutritional regulation of cell growth, mediates phosphorylation to activate downstream signaling pathways, transmits external stimulus signals, regulates physiological processes such as intracellular ribogenesis, protein synthesis, lipid synthesis, and energy metabolism, and integrally regulates cell growth, proliferation, apoptosis, and autophagy to achieve precise regulation of life processes^11^.

The TOR signaling pathway in model organisms represented by yeast, *Drosophila melanogaster*, *Caenorhabditis elegans*, and mice has been a hot research topic^12^. In contrast however, the TOR signaling pathway has been less studied in aquatic animals and has only been reported in a few fish species, including *Danio rerio*^13,14^, *Oncorhynchus mykiss*^15,16^, and *Cyprinus carpio*^17^, and its study in crustaceans is even rarer. *Macrobrachium rosenbergii* is a high market-value crustacean that is farmed globally, mainly in fresh or brackish waters, with the advantages of rapid growth and high nutritional value^18^. Given the lack of research on the molecular regulatory mechanisms of crustacean nutrition and metabolism, the present study aimed to analyze the expression characteristics of the *TOR* gene and signaling pathway in *M. rosenbergii* after different treatments. The results will improve our understanding of the molecular mechanisms regulating growth and metabolism in economically important crustaceans, and provide a theoretical basis for seedling selection and breeding, feed-nutrient additives and proportions, and optimization of culture management strategies.

## Results

### MrTOR sequence characteristics

**Figure 1:**
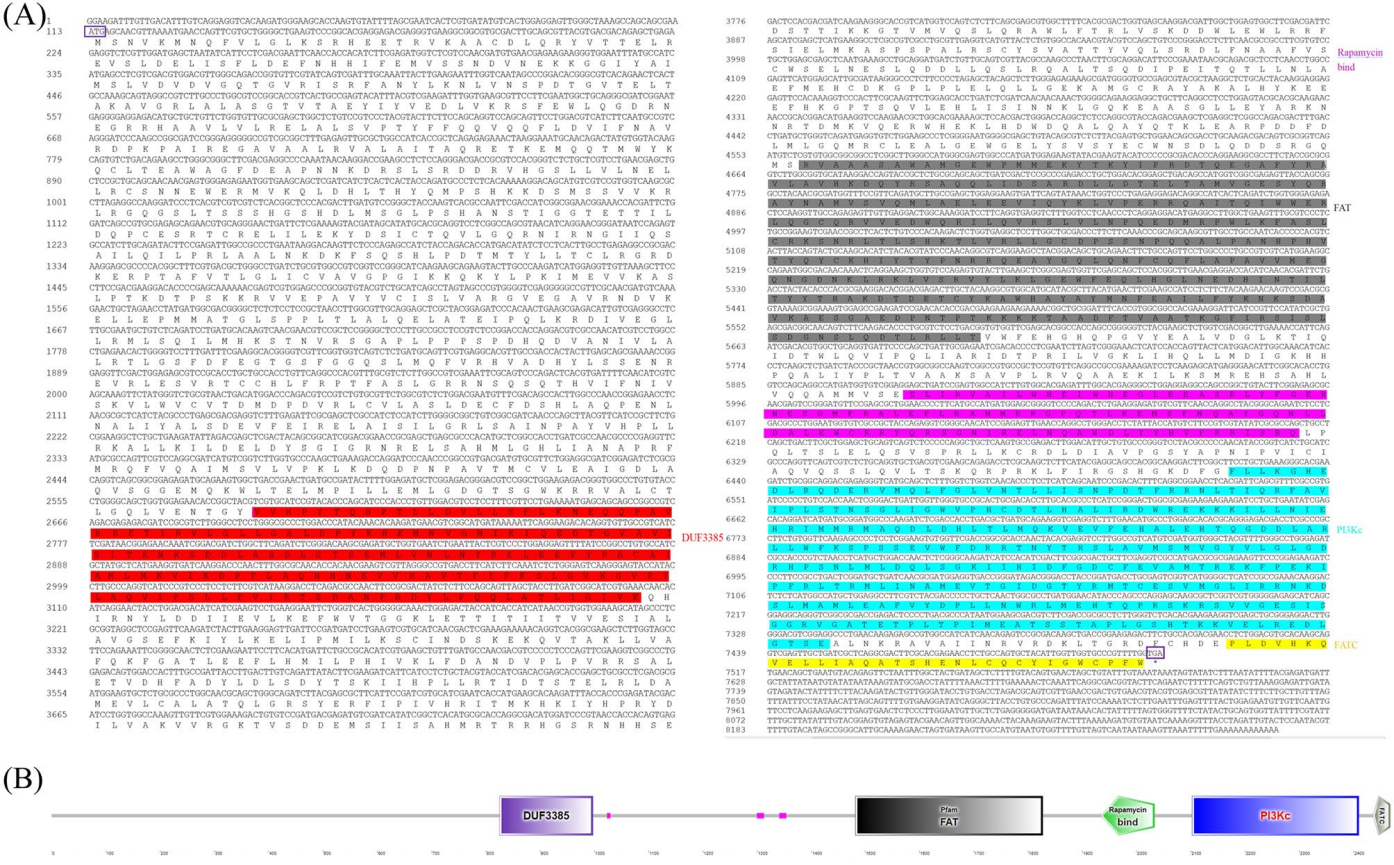
(**A**) Nucleotide and predicted amino acid sequences of *MrTOR*. Purple boxes indicate start (ATG) and stop codons (TGA). Red region represents DUF3385 structural domain; gray region, FAT structural domain; pink region, rapamycin-binding structural domain; blue region, PI3Kc structural domain; yellow region, FATC structural domain. (**B**) Predicted TOR protein functional domains.

We obtained a 8280 bp full-length cDNA of the *MrTOR* gene by RACE cloning, with a 3'-UTR of 764 bp, 5'-UTR of 112 bp, and an ORF of 7404 bp, encoding 2467 amino acids (Fig. 1A). The amino acid sequence of this gene contained five structural domains: the DUF3385 structural domain (residues 825–997), FAT structural domain (residues 1482–1827), rapamycin-binding structural domain (residues 1934–2033), phosphoinositide 3-kinase (PI3Kc) structural domain, residues 2102–2409), and the FATC structural domain (residues 2435–2467) (Fig. 1B). The protein had a predicted molecular weight of 280.424 kDa and a theoretical isoelectric point of 7.31, with no predicted transmembrane structure or signal peptide. Leu was the most abundant amino acid, accounting for 12.4% of the total amino acid content, followed by alanine, accounting for 7.2% of the total amino acid content. The GenBank accession number was OR358841.

**Figure 2:**
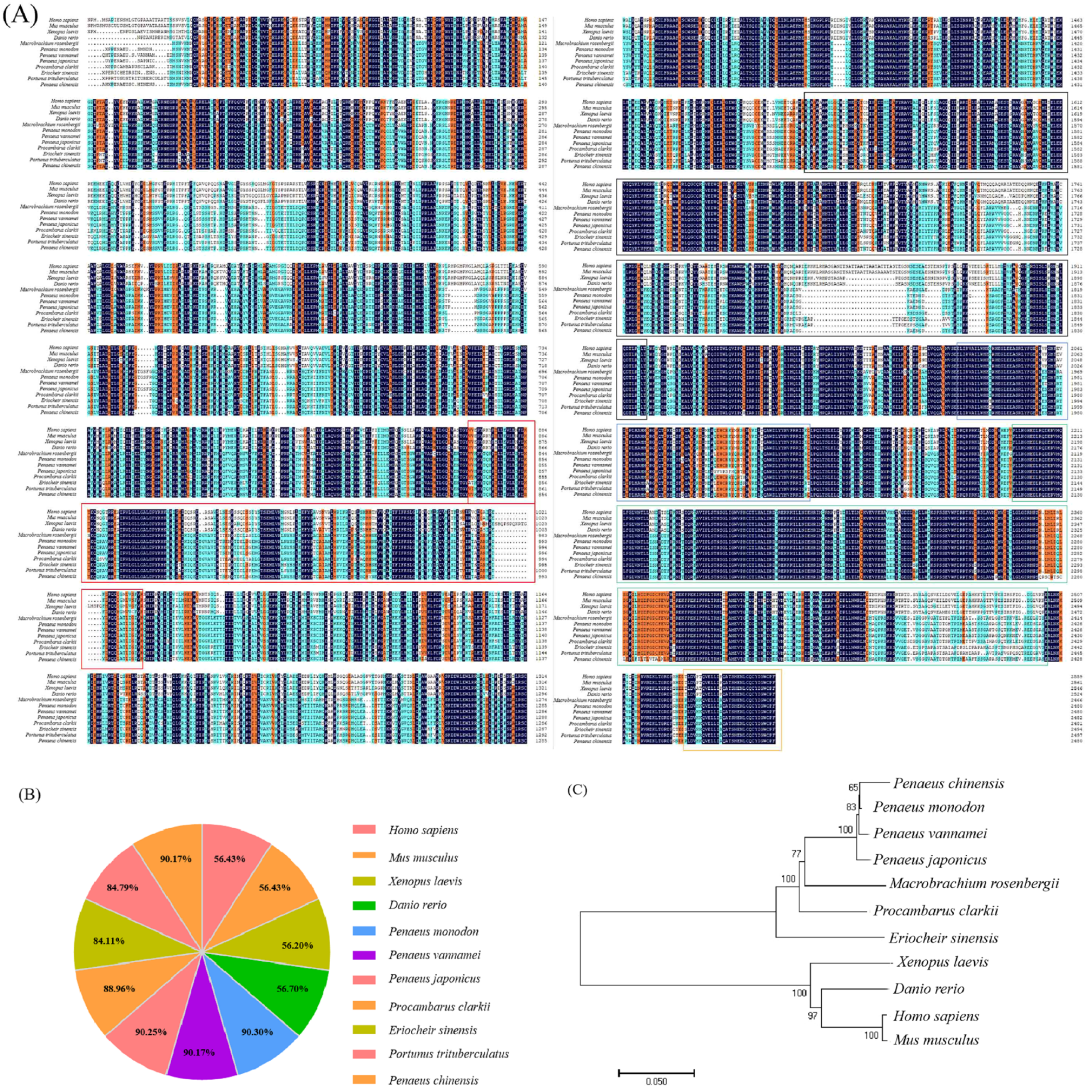
(**A**) Amino acid sequence comparison of MrTOR with other species. Red box indicates DUF3385 structural domain; black box, FAT structural domain; blue box, rapamycin-binding structural domain; green box, PI3Kc structural domain; orange box, FATC structural domain. GenBank accession numbers: *Homo sapiens* (NP_001373429.1); *Mus musculus* (NP_064393.2); *Xenopus laevis* (XP_018081150.1); *Danio rerio* (NP_001070679.2; *P. monodon* (XP_037804060.1); *Penaeus vannamei* (QHT73480.1); *P. japonicus* (UYO77156.1); *P. clarkii* (XP_045619169.1); *E. sinensis* (XP_050711953.1); *Portunus trituberculatus* (XP_045121188.1); *P. chinensis* (AHX84170.1). (**B**) Similarity of MrTOR amino acid sequence to TOR amino acid sequences of other species. (**C**) MrTOR phylogenetic analysis. The number on the node indicates the confidence value of the test for 1000 bootstrap repetitions.

The TOR amino acid sequences of 11 different species were selected from the NCBI database for homology comparison. The highest amino acid similarity of 90.3% was found between *M. rosenbergii* and *Penaeus monodon*, followed by *Penaeus japonicus*, *Penaeus chinensis*, and *Penaeus vannamei*, with amino acid similarities of 90.25%, 90.17% and 90.17%, respectively. Amino acid similarity with vertebrates was low (56.43% with *Homo sapiens*) (Fig. 2A,B).

The phylogenetic tree showed that vertebrates clustered into one unit and invertebrates into another. The shrimp family first clustered into one tribe, and then clustered with *M. rosenbergii*. *M. rosenbergii* was also closely related to the crustaceans *Procambarus clarkii* and *Eriocheir sinensis* (Fig. 2C). The evolutionary position of *MrTOR* in the phylogenetic tree was consistent with the taxonomic position.

### *MrTOR distribution in shrimp tissues and *in situ* hybridization*

**Figure 3:**
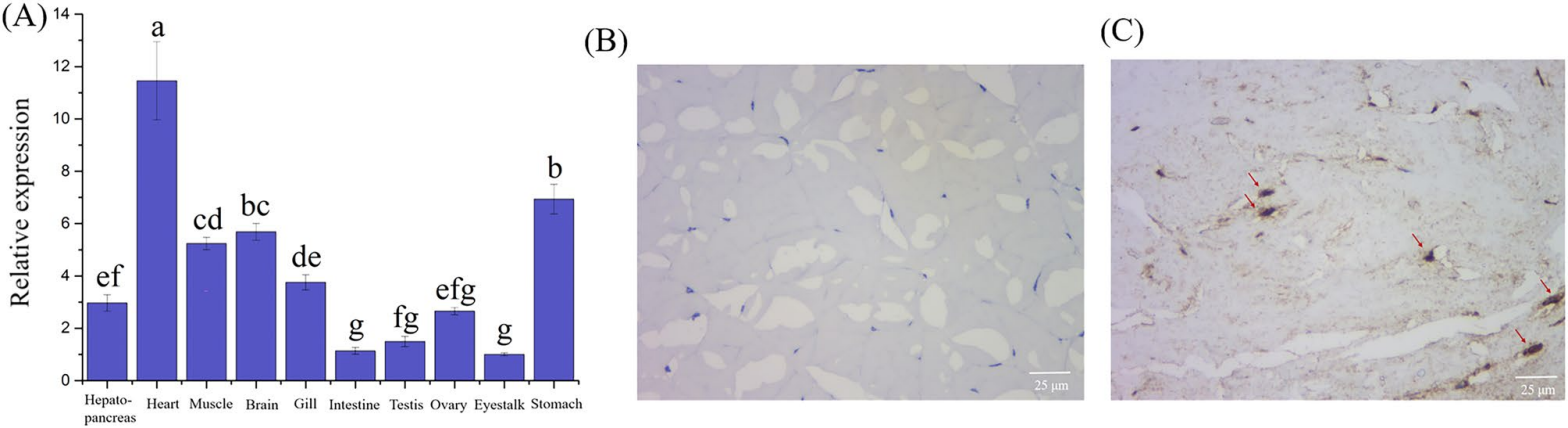
(**A**) *MrTO*R expression levels in each tissue. Different letters indicate significant differences (*P* < 0.05). Results after hybridization with sense probes (negative control). (**B**) and antisense probes (experimental group) (**C**). Arrows indicate positive signals.

Melting curve analysis revealed a peak of specificity in all genes. The amplification efficiency of all genes ranged from 91.0 to 109.4%. None of the primer pairs was amplified in the non-template control. *MrTOR* was expressed in all tissues in *M. rosenbergii* according to qRT-PCR, with lower expression in intestine and eyestalk tissues and higher expression in heart, stomach, and muscle tissues. The highest expression was in the heart, at 11.5-fold the expression level in the intestine (Fig. 3A). These results indicated that *MrTOR* gene expression was tissue-specific.

We further investigated the function of *MrTOR* in organismal nutrition by locating the expression of the *MrTOR* gene in muscle tissue. In situ hybridization showed that *MrTOR* was expressed in muscle, especially around the nucleus. No signal was detected in the control group (Fig. 3B,C).

### Effects of RNAi on TOR signaling pathway

**Figure 4:**
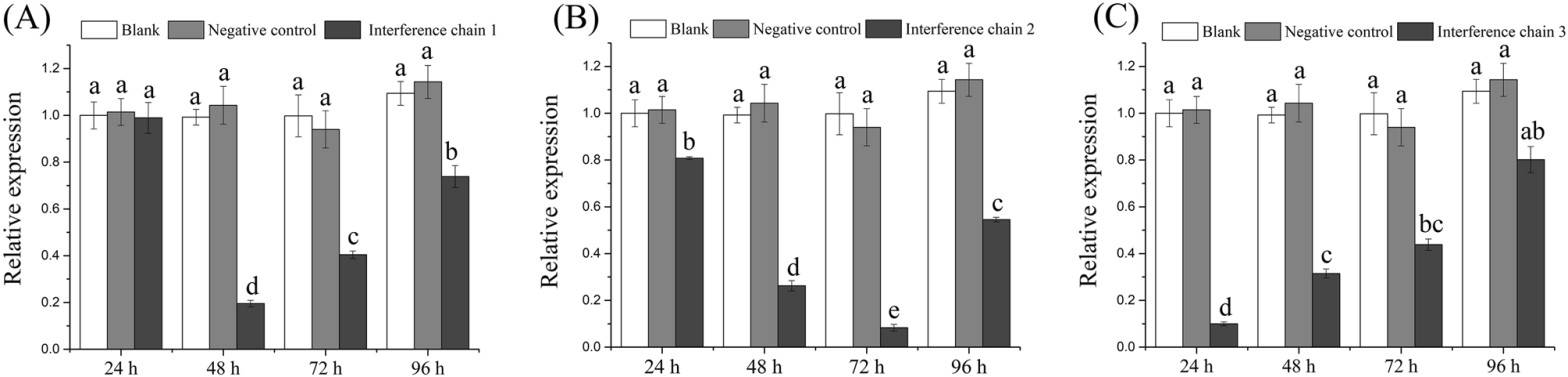
Relative expression levels of *MrTOR* in muscle after interference. Interference chain 1, 2 and 3 indicate G1, G2 and G3, respectively. Different letters indicate significant differences (*P* < 0.05).

The silencing efficiency of *MrTOR* was detected by qRT-PCR. All three siRNAs effectively reduced the expression of *MrTOR* (*P* < 0.05). G1 significantly down-regulated *MrTOR* mRNA levels 48 h after injection by 81% compared with the negative control group. The optimal time of interference for G3 was 24 h post-injection, with an inhibitory efficiency of 90%, while G2 inhibited *MrTOR* by 74% and 93% after 48 and 72 h, respectively (Fig. 4). G2 was therefore used for further RNAi experiments.

**Figure 5:**
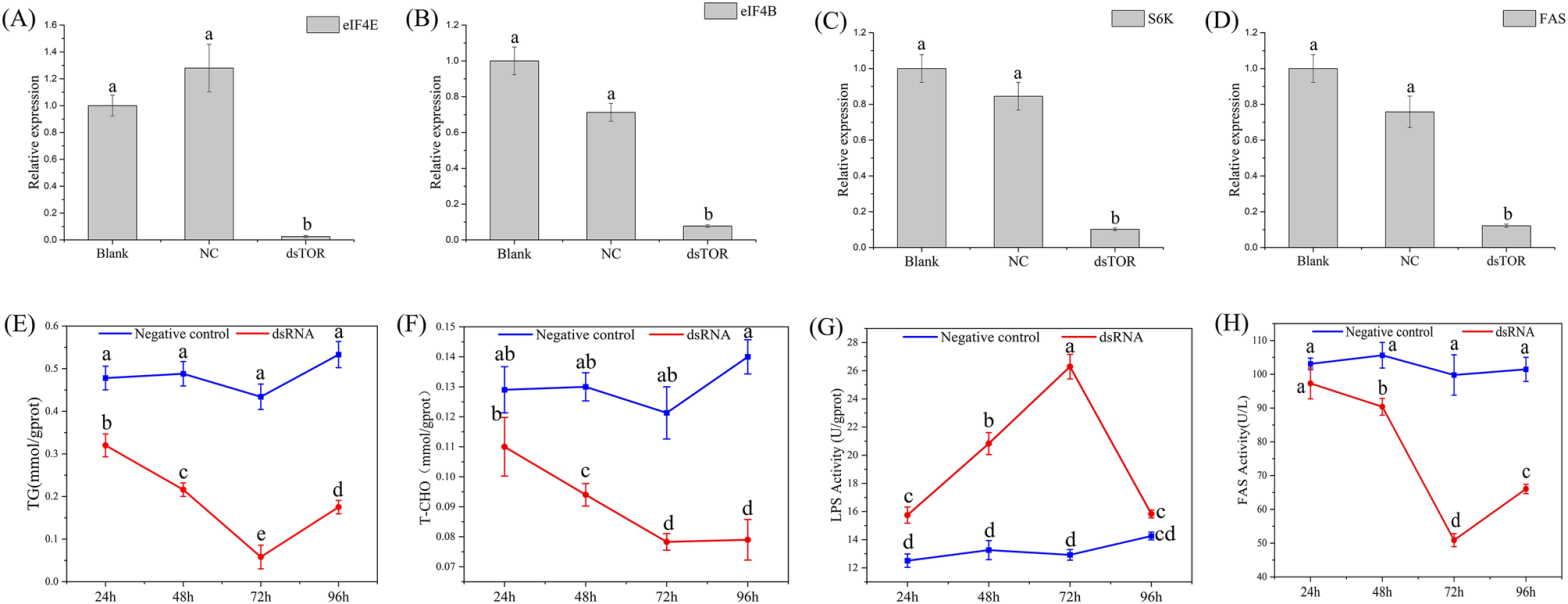
Relative expression levels of (**A**) *eIF4E*, (**B**) *eIF4B*, (**C**) *S6K*, and (**D**) *FAS* genes after interference. (**E**) TG and (**F**) T-CHO contents in tissue after interference. (**G**) LPS activity and (**H**) FAS activity after interference. Different letters indicate significant differences (*P* < 0.05).

To determine the potential role of the TOR signaling pathway in the regulation of muscle protein synthesis and lipid metabolism-related gene expression, we examined the expression levels of three protein synthesis-related genes (translation initiation factor 4E, *eIF4E*; eukaryotic translation initiation factor 4B, *eIF4B*; ribosomal protein S6 kinase, *S6K*) and one lipid synthesis-related gene (fatty acid synthase, *FAS*) after silencing *MrTOR*. The expression levels of *eIF4E*, *eIF4E*, *S6K*, and *FAS* were all significantly reduced after silencing *MrTOR* compared with the control and blank groups (*P* < 0.05; Fig. 5A–D).

We confirmed these results by examining the T-CHO and TG contents and LPS and FAS activities in muscle tissue after *MrTOR* silencing. The T-CHO and TG contents decreased 1.5-fold and ninefold, respectively, after 72 h of silencing (Fig. 5E,F), while LPS activity increased to 2.1-fold that in the control group and FAS activity decreased 2.3-fold (Fig. 5G,H). These results showed that silencing *MrTOR* significantly reduced FAS activity, increased LPS activity, and accelerated fat metabolism, leading to reductions in the T-CHO and TG contents.

### Effects of Leu and Arg on TOR signaling pathway

**Figure 6:**
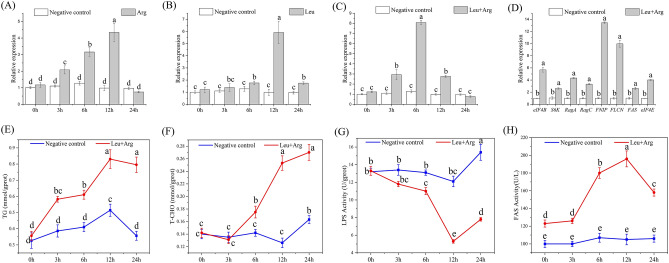
*MrTOR* expression levels after (**A**) Arg injection, (**B**) Leu injection, and (**C**) Leu and Arg injection. (**D**) Expression levels of different genes after amino acid injection. (**E**) TG and (**F**) T-CHO contents, and (**G**) LPS activity and (**H**) FAS activities at different times after amino acid injection. Different letters indicate significant differences (*P* < 0.05).

To investigate how the TOR signaling pathway functioned after receiving extracellular amino acid signals, we injected shrimps intraperitoneally with Leu and Arg, which are sensitive to TOR signaling. Injection of either amino acid increased *MrTOR* gene expression levels. *MrTOR* gene expression was significantly increased after 3 h of Arg injection (*P* < 0.05), and was increased 4.3-fold compared with the control group at 12 h after injection (Fig. 6A). Leu injection had no significant effect (*P* > 0.05) within 6 h, but *MrTOR* gene expression was significantly increased compared with the control group at 12 h after injection (*P* < 0.05) (Fig. 6B). *MrTOR* expression levels were significantly increased at 3 h after injection of equimolar ratios of Arg and Leu compared with the control group (*P* < 0.05), and expression levels were increased 8.1-fold at 6 h after injection (Fig. 6C). Injection of equimolar ratios of Arg and Leu thus activated *MrTOR* gene expression more than injection of either amino acid alone.

We detected expression levels of *MrTOR* signaling pathway-related genes in tissue samples injected with equimolar ratios of Arg and Leu for 6 h. The relative expression levels of eight genes were detected by qRT-PCR: *eIF4B*, *S6K*, *eIF4E*, Ras-related GTP-binding protein A (*RagA*), Ras-related GTP-binding protein C (*RagC*), folliculin (*FLCN*), folliculin-interacting protein (*FNIP*), and *FAS*. Expression levels of all these genes were significantly increased after amino acid injection, with expression levels of *FNIP* and *FLCN* up-regulated 13.3-fold and 10.1-fold, respectively (Fig. 6D). The T-CHO and TG contents in muscle tissue showed increasing trends after amino acid injection (Fig. 6E,F). LPS activity decreased significantly after 6 h of injection (*P* < 0.05) and decreased 2.5-fold by 12 h (Fig. 6G), while FAS activity increased significantly after injection (*P* < 0.05) (Fig. 6H). These results indicated that amino acid injection could accelerate the processes of lipid and protein synthesis.

### Effects of starvation and post-starvation amino acid injection on TOR signaling pathway

**Figure 7:**
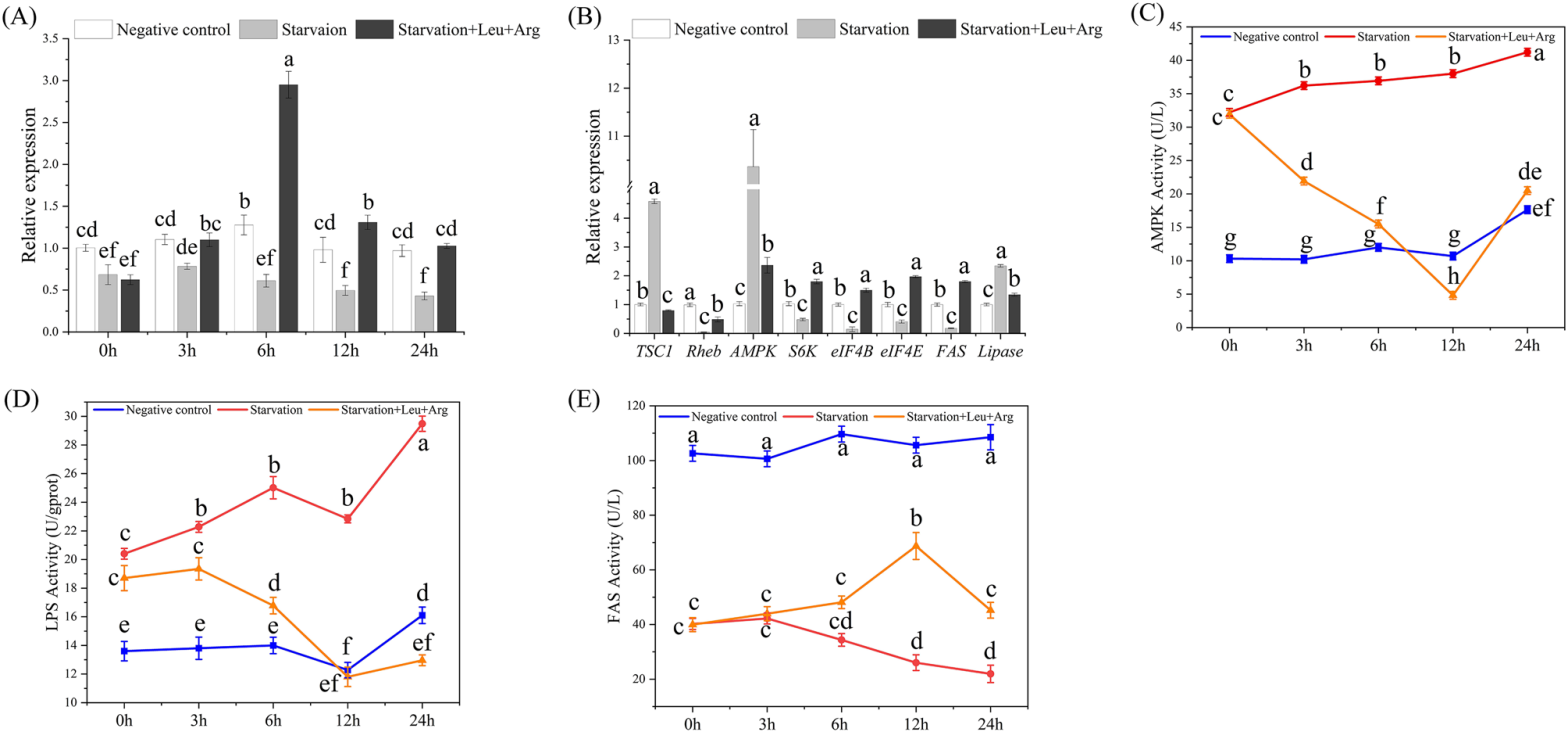
Expression levels of (**A**) *MrTOR* and (**B**) amino acid-related genes after starvation and post-starvation amino acid injection. (**C**) AMPK, (**D**) LPS, and (**E**) FAS activities after starvation and post-starvation amino acid injection. Different letters indicate significant differences (*P* < 0.05).

*MrTOR* gene expression was significantly decreased after 3 days of starvation (*P* < 0.05), whereas injection of Leu and Arg increased the expression of *MrTOR*, peaking after 6 h (Fig. 7A). We examined the expression levels of the relevant genes after 6 h of amino acid injection, and showed that mRNA expression levels of tuberous sclerosis complex 1 *(TSC1)*, *AMPK*, and *LPS* were upregulated by starvation treatment compared with the control group, whereas expression levels of *Rheb* and protein synthesis-related genes (*eIF4B*, *S6K*, *eIF4E*, and *FAS*) were down-regulated. Amino acid injection significantly reduced expression levels of *TSC1*, *AMPK*, and *LPS* compared with the starvation group, (*P* < 0.05), and significantly increased expression levels of *Rheb*, *eIF4B*, *eIF4E*, and *S6K* (*P* < 0.05, Fig. 7B). AMPK and LPS activities tended to increase gradually with duration of starvation, while FAS enzyme activity tended to decrease. Amino acid injection significantly reduced AMPK and LPS activities (*P* < 0.05), which both reached their lowest levels at 12 h (Fig. 7C,D), while FAS activity gradually increased and peaked at 12 h (Fig. 7E). These enzyme activity results were consistent with the gene expression levels, suggesting that injection of Arg and Leu could effectively abrogate starvation-induced changes in the TOR pathway.

## Discussion

TOR converges and integrates stimulatory signals from environmental stresses to regulate cell growth via downstream effectors^19^, and also responds to extracellular stimuli including amino acids, glucose, and growth factors. TOR is thus a key factor in growth regulation and exists in a wide range of cells^20^. In this study, we identified and isolated the *TOR* gene from *M. rosenbergii and* showed that it included DUF3385, FAT, rapamycin-binding, PI3KC, and FATC structural domains. The DUF3385 structural domain is an unknown functional domain that is widely found in eukaryotes^21^. The rapamycin-binding domain is a key site where rapamycin binds to TOR, thereby inhibiting TOR protein function. Mutations in this region prevent the inhibitory effect of rapamycin on TOR^22^. The sequence of the PI3KC kinase structural domain of the TOR protein is highly homologous to the kinase catalytic domain of PI3K, contains most of the active site, and has fully ordered structural elements essential for catalysis^23^. The FAT and terminal FATC structural domains can form a spatial structure in which FAT/FATC bind to each other, exposing the TOR catalytic structural domain and participating in the interaction of TOR molecules^24^. Each part of the TOR protein plays an important role in its function, and changes in any of these regions can affect TOR activity^25^. The amino acid sequence of *MrTOR* showed high similarity with that in shrimps such as *P. monodon*, indicating that the biological function of *MrTOR* was relatively conserved. Analysis of the phylogenetic tree showed that *M. rosenbergii* was more closely related to *P. trituberculatus* than to *E. sinensis*, which is a crustacean in the same genus. The location of *M. rosenbergii* on the same large branch as other shrimps also indicated that *MrTOR* was relatively conserved.

Quantitative results showed that *MrTOR* was expressed in all tissues, indicating that it was involved in the regulation of multiple physiological functions in the organism. However, expression levels varied among tissues, with the highest expression in the heart. The mammalian TOR (mTOR) pathway has been shown to play a role in cardiac development and disease in children^26^, suggesting that MrTOR proteins might also be involved in cardiac development in larval *M. rosenbergii*. The mTOR pathway has been reported to regulate nutrient metabolism and utilization in skeletal muscle cells and promote muscle protein synthesis. mTOR is a key regulator of skeletal muscle development by controlling different stages of myogenesis^27–29^. The current quantitative results showed that *MrTOR* was highly expressed in muscle tissue, and in situ hybridization showed that it was expressed in muscle tissues, especially around the nucleus. We therefore hypothesized that MrTOR plays an important role in muscle protein synthesis in *M. rosenbergii*.

The most prominent downstream target proteins of mTOR are S6K1 and 4EBP1, which are positive and negative regulators of protein synthesis, respectively. Phosphorylated S6K1 further phosphorylates multiple downstream substrates to regulate intracellular translation, including activation of eIF4B or S6, which activates translation initiation and ribosome biosynthesis^30,31^. Phosphorylation of 4EBP1 by mTOR leads to its detachment from eIF4E, allowing eIF4E to bind to factors such as eIF4G to form a translation initiation complex. The mTOR signaling thus promotes intracellular protein translation, ribosome biosynthesis, and cell growth via the S6K and 4EBP1 pathways^32^. In the current study, expression levels of *MrTOR* were significantly reduced by injection of siRNA, and expression levels of *S6K*, *eIF4B*, and *eIF4E* were also significantly reduced after knockdown of *MrTOR*. We hypothesized that MrTOR had similar a biological function in *M. rosenbergii* to that in mammals, and that it activated the expression of the downstream effectors S6K and eIF4E, and promoted protein synthesis and cell growth. In addition to regulating protein synthesis, TORC1 also regulates lipid synthesis and has been reported to activate the transcription factor SREBP via two pathways, S6K1 and Lipin1, to regulate the transcription of fatty acid- and cholesterol-related metabolic genes and promote fatty acid synthesis^33,34^. Accumulating evidence also suggests that inhibition of mTOR expression significantly attenuated hepatic lipid accumulation in rats fed a high fat diet^35,36^, while knockdown of S6K1 in mice enhanced lipolysis levels and increased energy expenditure without obesity^37,38^. In the present study, knockdown of *MrTOR* decreased *FAS* expression and enzyme activity, increased LPS enzyme activity, and significantly reduced TG and T-CHO levels in tissues. These results suggested that inhibiting the expression of *MrTOR* inhibited lipid synthesis and accelerates lipid catabolism.

Most studies of TOR have focused on its participation in cell growth and nutritional regulation. Amino acids are considered to act directly as positive regulators of the TOR signaling pathway, and the deletion of various individual amino acids, such as Arg, from allogeneic cultures showed inhibitory effects on the TOR signaling pathway^39^. Activation of the TOR signaling pathway by Leu and Arg was also found to be most pronounced when a single amino acid was added to amino acid-free cell cultures^40^. Leu can stimulate protein synthesis through a number of factors that initiate mRNA translation, mainly by activating the mTOR signaling pathway, including S6K1 and 4EBP1, leading to increased levels of phosphorylation and thereby regulating the expression of ingestive metabolism genes at either the transcriptional or translational level^41^. In this study, we observed the effects of injecting Leu, Arg, and an equimolar mixture of the two amino acids on *MrTOR* expression in the muscle tissue in *M. rosenbergii*, as well as their effects on the TOR signaling pathway. Our results revealed that *MrTOR* expression was significantly up-regulated by amino acid injection, and that injection of equimolar ratios of Arg and Leu had a greater effect than injection of either amino acid alone. Transcript levels of downstream related genes (*S6K*, *eIF4E*, *eIF4B*, and *FAS*) were also significantly increased while T-CHO and TG levels in tissues, as well as FAS enzyme activity, were significantly elevated in line with the above findings. These results suggested that amino acid treatment could activate the TOR signaling pathway and promote intracellular protein and lipid syntheses. The addition of amino acids has been reported to promote the binding of mTORC1 to Rag GTPases (consisting of RagA/B and RagC/D), which in turn activates mTORC1^42^. FLCN, as a tumor suppressor, is usually involved in the mTORC1 cell signaling pathway together with FNIP. FLCN-FNIP can act as GAP-activated Rags of RagC/D and are free in the cytoplasm to play a positive regulatory role in the mTORC1 signaling pathway^43^. The current results showed that the expression levels of *FNIP*, *FLCN*, *RagA*, and *RagC* were all up-regulated after amino acid injection. We therefore hypothesized that these genes might also be involved in the amino acid to TOR signaling process; however, further studies are needed to determine the exact mechanism by which the amino acid signals are passed on to TORC1. The nutritional and energetic state of the cell is signaled to TOR via AMPK, a serine/threonine kinase that is present in all eukaryotes as a key energy receptor^44^. During nutrient deprivation, AMPK directly phosphorylates TSC1/2 and Raptor proteins to deliver signals to TORC1, thus preventing cell growth and conserving energy^45^. *Rheb* is a downstream target gene of TSC1/2 that antagonizes the mTOR endogenous inhibitor FKBP 38 in a guanosine triphosphate-dependent manner and binds directly to the carboxyl terminus of the mTOR catalytic domain and regulates the kinase activity with the participation of phospholipase D1^46^. Starvation inhibits TOR pathway activity, and overexpression of Rheb mitigates starvation-induced changes^47,48^. In this study, *MrTOR* expression was significantly suppressed after 3 days of starvation, consistent with the above results. In addition, the transcript levels and enzyme activities of *AMPK* and *LPS* were significantly increased after starvation, transcript levels and enzyme activities of *FAS* were significantly decreased, and the transcript level of *TSC1* was down-regulated, whereas the transcript level of *Rheb* was up-regulated, suggesting that *AMPK* was activated by starvation to up-regulate the expression of *TSC1*, thereby inhibiting the transcription of *Rheb*, decreasing the expression of *TOR*, blocking the anabolic metabolism of proteins and lipids, and promoting lipid catabolism to provide energy for the cells. The current experiments also explored the effect of amino acid injection on starvation-induced changes in the pathway, and showed that amino acid injection could effectively alleviate the low expression of *MrTOR* induced by starvation, suggesting that regulation of the TOR pathway by amino acids was important in growth and metabolism in the organism.

## Conclusion

**Figure 8:**
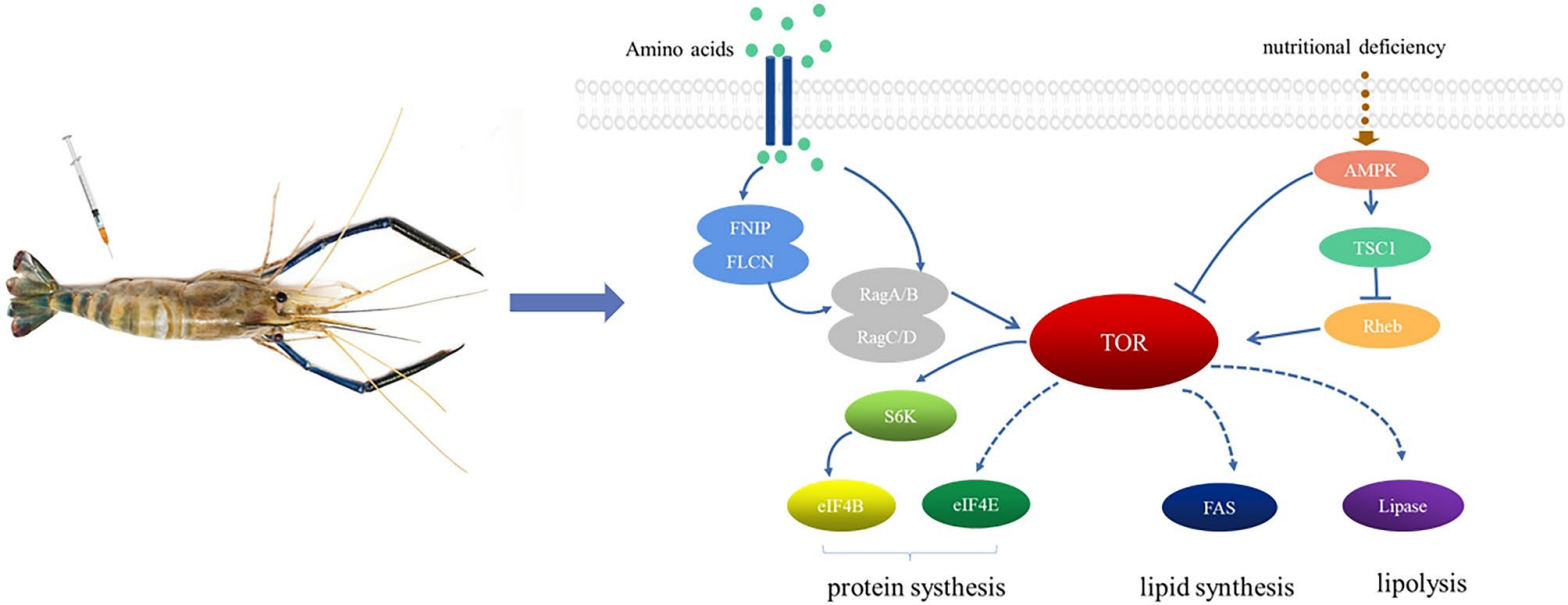
Predicted TOR signaling pathway in *M. rosenbergii.*

In conclusion, we cloned and characterized the full-length sequence of *MrTOR* from *M. rosenbergii*. The amino acid sequence showed high similarity with those of other crustaceans, suggesting that the biological functions of MrTOR were conserved. Tissue expression profiling and in situ hybridization showed high expression levels of *MrTOR* in tissues such as the heart and muscle, suggesting that it might play an important role in larval heart development and muscle protein synthesis. RNAi results showed that *MrTOR* activated the expression of the downstream effectors *S6K* and *eIF4E* to promote protein synthesis, and inhibition of *MrTOR* expression could inhibit lipid synthesis and accelerate lipid catabolism. The TOR signaling pathway was activated by amino acid treatment to promote intracellular protein and lipid synthesis, and injections of equimolar ratios of Arg and Leu were more effective than either amino acid alone. Starvation could activate AMPK, which transmits signals to TOR and promotes lipid catabolism to provide energy for cells. Amino acids could effectively mitigate starvation-induced changes in the TOR signaling pathway (Fig. 8).

## Materials and methods

### Experimental animals and sample collection

Healthy *M. rosenbergii* (body length 5.0 ± 1.0 cm, body weight 2.0 ± 0.5 g) were purchased from Shanghai Shencao Special Aquatic Products Development Co Ltd., and were of uniform specification with no body surface damage. The experimental animals were temporarily cultured in 100-L tanks at 27.0 ± 1.0 °C, pH 7.5–8.2, dissolved oxygen 5.0–6.5 mg/L, 24 h continuous aeration, 50% daily water change, and natural photoperiod. Before the experiments, the animals were reared temporarily for 7 days to adapt to the indoor culture environment.

Tissues including gills, hepatopancreas, gonads, muscles, heart, brain, intestines, and eye stalks were dissected from the shrimps using sterile scissors and forceps, frozen rapidly in liquid nitrogen, and stored at −80 °C for RNA extraction.

### Total RNA extraction and cDNA synthesis

RNA was extracted from tissue samples using TRIzol (TaKaRa, Shiga, Japan), and the concentration and purity of the extracted RNA were determined using a NanoDrop 2000C spectrophotometer (Thermo Fisher Scientific, MA, USA). The integrity of the RNA was verified by 1% agarose gel electrophoresis and the RNA was then reverse transcribed into cDNA using a Hifair® V Reverse Transcriptase Reverse Transcription Premix Kit (Yeasen Biotechnology, Shanghai, China).

### Full-length cloning of cDNA

Rapid amplification was performed using SMART 5' and 3′ rapid amplification of cDNA ends (RACE) kits (TaKaRa). Primers based on partial transcriptome sequences were designed using Primer 5.0 (Table 1) and submitted to GENEWIZ (Suzhou, China) for synthesis. The amplification products were purified using a MiniBEST Agarose Gel DNA Extraction Kit Ver.4.0 (TaKaRa). The recovered purified products were ligated into linear vectors under the following ligation conditions: overnight ligation at 16 °C with 5 µL of the recovered product, 1 µL of PMD19-T Easy, and 4 µL of Solution I. The ligation product was then added to DH5α and subjected to heat stimulation, and liquid medium was then added and incubated on a shaker for 2 h. The plates were coated and placed in an incubator at 37 °C overnight, and single colonies were then picked into LB liquid medium for amplification. Finally, positive clones were selected and sent to GENEWIZ for sequencing. We spliced the sequencing results to obtain the full-length sequence of *TOR*, named *MrTOR*.

**Table 1:** Primers used in the experiment.

Primer Name	Sequence (5´to 3´)	Purpose
*MrTOR*	F: TGGACCACTTTGCCGATTA R: GACGCCTGTTGGGATACG	partial sequence PCR
*MrTOR-5’*	F: TCCCAGTCGTGGAGCTTTTC R: AGGGCTATGCTTTCCACCAC	5´RACE
*MrTOR-3’*	F: TCCCACAACTGAAGCGAGAC R: TCGTGGAAAGACTGTGTCCG	3´RACE
*RT-MrTOR*	F: TCCCACAACTGAAGCGAGAC R: AGTCGAACCTCCCGGTTTTC	qRT-PCR
*18S*	F: GCTCTTTACCGAGTGTCCC R: TTCGCTGTTGTTCGTCCTA	Reference gene
*SiRNA1-MrTOR*	F: GCCCAAGAUCAUGGAGGUUTT R: AACCUCCAUGAUCUUGGGCTT	DsRNA
*SiRNA2-MrTOR*	F: GCAGGACAUUCCCGAAAUATT R: UAUUUCGGGAAUGCCUGCTT	
*SiRNA3-MrTOR*	F: GCUGCGACCCUUCUUCAAATT R: UUUGAAGAAGGGUCGGAGCTT	
*MrTOR-ISH*	F: AACCGCCTCACTCTGTCCCA R: CCTGAAAGTGTCGGGATTGCT	In situ hybridization probe
*FAS*	F: TTCAATACGGTGGTGTCC R: AAGTGCGATGTCATACGG	Related genes
*AMPK*	F: CTACCTCGTGGAGAAGCAGA R: AATACAGCCGTCGTAATCG	
*eIF4E*	F: AAGGCGGCAGATTTGTAT R: TCTCCACTCGAAAGCAGT	
*S6K1*	F: CACTACGCTTTCCAGACC R: TCCAATGCTAAGGCTAAT	
*eIF4B*	F: ACCACCTGCTACATCAATAC R: CCTTTCACGGCTTACATT	
*Lipase*	F: CCACCATTGTCTCCTCTGC R: CGTGCCAACTGAACTCCC	
*TSC1*	F: TGTTCGTCTCCACCCTAT R: AAATTCATTTCCGCCACC	
*RagA*	F: GTCATCTCCCATTATCGC R: GTGCTTTCGTGCATTCTT	
*FNIP*	F: AGGTGCTGCTGGGAGTGA R: TTGCCGTTCGTTGTTGTG	
*FLCN*	F: TACCATCCCGTCCTCCAC R: AACCCAAACCTTCACCTTATT	
*RagC*	F: TTTACGGGAAAGATGAAG R: TGCCAAATACCGACTGAC	
*Rheb*	F: TTTCCTGCTCACTATTCTAT R: ATCATTCTTGTTACCCACTA	

### Bioinformatics analysis

Open reading frame (ORF) and amino acid sequences were predicted from the resulting sequences using the NCBI online analysis tool ORF Finder (http://www.ncbi.nlm.nih.gov/gorf/gorf.html). Physical parameters were analyzed with Protparam in ExPasy (http://www.expasy.org/tools). Transmembrane structures and signal peptides were predicted using TMHMM Server v2.0 (http://www.cbs.dtu.dk/services/TMHMM/) and SignalP-5.0 (http://www.cbs.dtu.dk/services/SignalP/) and structural domains were predicted using SMART (http. //smart.embl-heidelberg.de/). Amino acid sequences were compared using DNAMAN 9.0, and phylogenetic trees were constructed using the MEGA 7.0 neighbor-joining method.

### Quantitative real-time polymerase chain reaction (qRT-PCR) to detect MrTOR expression

The ORF region was used to design qRT-PCR-specific primers (Table 1). A standard curve for each primer was created using a linear regression equation with tenfold serial dilutions of cDNA as the template. Amplification was performed in triplicate on a Bio-Rad CFX96 (Bio-Rad, Hercules, CA, USA) using Hieff® qPCR SYBR Green Master Mix (Yeasen Biotechnology, Shanghai). A non-template control was also included with each primer pair. The reaction system was as follows: 10 µL 2 × SYBR Green Master Mix, 0.4 µL forward primer (10 µmol/L), 0.4 µL reverse primer (10 µmol/L), 7.2 µL ddH_2_O, and 2 µL cDNA (equivalent to 100 ng of total RNA). The amplification conditions were: 95 °C for 2 min, 95 °C for 10 s, 60 °C for 30 s, and 40 cycles. Fluorescence signals were gathered for the lysis curve throughout the extension phase, with each cycle lasting 5 s for every 0.5 °C rise from 65 to 95 °C. Data analysis was carried out with three biological and three technical replicates. The amplification results were analyzed by the 2^−∆∆CT^ method to obtain the expression of each sample relative to the internal reference gene 18S^49^.

### In situ* hybridization*

Specific primers were designed using Primer 5.0 (Table 1), and linear DNA with T7 and SP6 promoters at both ends was amplified as templates. In vitro transcription to synthesize DIG-labeled sense and antisense probes, respectively, was carried out a DIG RNA labeling kit (SP6/T7; Roche, Germany) and the probes were purified.

Muscle tissues were fixed in 4% paraformaldehyde, gradient dehydrated, embedded, sectioned, and then hybridized using a DIG Nucleic Acid Detection Kit (SP6/T7; Roche, according to the manufacturer’s instructions. After DAB color development, the tissues were photographed and observed using a Nikon AZ100 microscope (Nikon, Japan).

### Small interfering RNA (siRNA) synthesis and injection

Three pairs of siRNA primers (G1, G2, and G3) were designed according to the ORF region (Table 1) and submitted to Sangon Biotech (Shanghai, China) for synthesis. A total of 240 healthy shrimps were randomly selected and divided equally into five groups: a blank group, a negative control group (injected with diethyl pyrocarbonate water), and three experimental groups (injected with siRNA). Animals were injected in the penultimate abdominal segment. According to the results of the pre-test, the siRNA was injected at a concentration of 1 µg/g. Muscle tissues were sampled 24, 48, 72, and 96 h after injection. Three parallel samples were taken from each experimental and control group, each of which was a mixed sample of muscle tissue from three shrimps. The samples were quickly placed in liquid nitrogen for freezing and stored at −80 °C for subsequent studies.

### Amino acid injections and starvation treatment

A total of 300 shrimps were selected randomly and divided into six groups: group 1 received 100 μL of phosphate-buffered saline (PBS; 0.01 M, pH 7.4) injected into the penultimate abdominal segment; groups 2 and 3 were injected with 100 μL leucine (Leu; 0.1 M) and arginine (Arg; 0.1 M), respectively, at the same location; group 4 was injected with 1:1 Leu (0.1 M) plus Arg (0.1 M); and groups 5 and 6 were first starved for 3 days and then injected. Injection concentrations were determined based on Hara and Kimball et al^50,39^. Based on the results for the first four groups, group 5 was injected with PBS at the same location and group 6 was injected with Leu and Arg in a molar ratio of 1:1. Samples were obtained at 0, 3, 6, 12, and 24 h. Muscle tissue was collected from nine shrimps from each experimental group at each time point. Tissue samples from every three shrimps were mixed. The samples were quickly placed in liquid nitrogen for freezing and stored at −80 °C for subsequent studies.

### Measurement of total cholesterol (T-CHO), triglycerides (TG), and enzyme activity

The tissues were weighed and saline was added at a weight (g):volume (mL) ratio of 1:9. The tissues were then homogenized mechanically in an ice-water bath, centrifuged at 12,000 rpm/min for 10 min, and the supernatant was collected for testing using T-CHO, TG, and lipase (LPS) assay kits, a shrimp adenylate-activated protein kinase (AMPK) enzyme immunoassay kit, and shrimp fatty acid synthase (FAS) enzyme immunoassay kit (Nanjing Jiancheng Bioengineering Institute, Nanjing, China), according to the manufacturer’s instructions.

### Statistical analyses

Statistical analysis was carried out using SPSS 22.0 and figures were created using Origin 9.1. Enzyme activity, qPCR, and tissue metabolite content results were expressed as mean ± standard error. Tukey’s method was used to analyze multiple comparisons using the results of one-way analysis of variance (ANOVA). Independent samples t-test was used to compare the significance of differences in relative expression between the two groups.

### Ethical approval and consent to participate

All animal procedures were performed in accordance with the Guidelines for the Care and Use of Laboratory Animals and approved by the Animal Ethics Committee of Shanghai Ocean University (Approval No. SHOU-DW-2022–051). All methods are in accordance with ARRIVE guidelines (https://arriveguidelines.org).

## Data Availability

All the data presented in this study are included in the article. If needed, supplementary material is available on request from the corresponding author.

## References

[1] Ai, C.X. Research progress in molecular nutriology of aquatic animal. *Fujian J. Agricult. Sci*. 10.19303/j.issn.1008-0384.2005.s1.013. (2005).

[2] Wang, J.Y. & Wang, X.H. The research development of molecular Nutriology. *Mod. Chem. Res.* 96–97. 10.3969/j.issn.1672-8114.2017.02.052. (2017).

[3] Sun, C.H. Development of nutrition science:retrospect and prospect. *Chin. J. Prev. Med.* 23–24. 10.3760/j:issn:0253-9624.2003.05.012. (2003).

[4] Heitman J, Movva NR, Hall MN (1991). Targets for cell cycle arrest by the immunosuppressant rapamycin in yeast. Science.

[5] Xin, F. *et al*. Mechanistic target of rapamycin signaling in aquatic animals. *Marine Sci.* **40**, 147–154. 10.11759/hykx20150115001. (2016).

[6] Oshiro N (2004). Dissociation of raptor from mTOR is a mechanism of rapamycin-induced inhibition of mTOR function. Genes Cells.

[7] Loewith R (2002). Two TOR complexes, only one of which is rapamycin sensitive, have distinct roles in cell growth control. Mol. Cell.

[8] Laplante M, Sabatini DM (2012). mTOR signaling in growth control and disease. Cell.

[9] Kim J, Guan KL (2011). Amino acid signaling in TOR activation. Annu Rev Biochem.

[10] Avruch J (2006). Insulin and amino-acid regulation of mTOR signaling and kinase activity through the Rheb GTPase. Oncogene.

[11] Cardenas ME, Cutler NS, Lorenz MC, Di Como CJ, Heitman J (1999). The TOR signaling cascade regulates gene expression in response to nutrients. Genes Dev..

[12] Neshat MS (2001). Enhanced sensitivity of PTEN-deficient tumors to inhibition of FRAP/mTOR. Proc. Natl. Acad. Sci. USA..

[13] Sapp V, Gaffney L, EauClaire SF, Matthews RP (2014). Fructose leads to hepatic steatosis in zebrafish that is reversed by mechanistic target of rapamycin (mTOR) inhibition. Hepatology.

[14] Ding Y, Sun X, Xu X (2012). TOR-autophagy signaling in adult zebrafish models of cardiomyopathy. Autophagy.

[15] Skiba-Cassy S, Lansard M, Panserat S, Medale F (2009). Rainbow trout genetically selected for greater muscle fat content display increased activation of liver TOR signaling and lipogenic gene expression. Am. J. Physiol. Regul. Integr. Comp. Physiol..

[16] Seiliez, I., Taty Taty, G.C., Bugeon, J., Dias, K., Sabin, N. & Gabillard, J.C. Myostatin induces atrophy of trout myotubes through inhibiting the TORC1 signaling and promoting Ubiquitin-Proteasome and Autophagy-Lysosome degradative pathways. *Gen. Comp. Endocrinol.* **186**, 9–15. 10.1016/j.ygcen.2013.02.008. (2013). 10.1016/j.ygcen.2013.02.008 23458288

[17] Wang B (2015). Effects of dietary arginine supplementation on growth performance, flesh quality, muscle antioxidant capacity and antioxidant-related signalling molecule expression in young grass carp (Ctenopharyngodon idella). Food Chem..

[18] Schwantes VS, Diana JS, Yi Y (2009). Social, economic, and production characteristics of giant river prawn Macrobrachium rosenbergii culture in Thailand. Aquaculture.

[19] Wullschleger S, Loewith R, Hall MN (2006). TOR signaling in growth and metabolism. Cell.

[20] Wang, Z.G., Wu, Y.J. & Xu, R.G. The mTOR signaling pathway and the regulation of cell growth. *Acta Biophysica Sinica*. 333–342. 10.3321/j.issn:1000-6737.2007.05.003. (2007).

[21] Marchler-Bauer A (2009). CDD: Specific functional annotation with the conserved domain database. Nucleic Acids Res..

[22] Sabatini DM, Erdjument-Bromage H, Lui M, Tempst P, Snyder SH (1994). RAFT1: a mammalian protein that binds to FKBP12 in a rapamycin-dependent fashion and is homologous to yeast TORs. Cell.

[23] Baretic D, Williams RL (2014). The structural basis for mTOR function. Semin. Cell Dev. Biol..

[24] Asnaghi L, Bruno P, Priulla M, Nicolin A (2004). mTOR: a protein kinase switching between life and death. Pharmacol. Res..

[25] Brown EJ (1994). A mammalian protein targeted by G1-arresting rapamycin-receptor complex. Nature.

[26] Kotulska K (2009). Cardiac rhabdomyomas in tuberous sclerosis complex show apoptosis regulation and mTOR pathway abnormalities. Pediatr. Dev. Pathol..

[27] Dickinson JM (2011). Mammalian target of rapamycin complex 1 activation is required for the stimulation of human skeletal muscle protein synthesis by essential amino acids. J. Nutr..

[28] McClung JP, Tarr TN, Barnes BR, Scrimgeour AG, Young AJ (2007). Effect of supplemental dietary zinc on the mammalian target of rapamycin (mTOR) signaling pathway in skeletal muscle and liver from post-absorptive mice. Biol. Trace Elem. Res..

[29] Norton LE (2009). The leucine content of a complete meal directs peak activation but not duration of skeletal muscle protein synthesis and mammalian target of rapamycin signaling in rats. J. Nutr..

[30] Holz MK, Ballif BA, Gygi SP, Blenis J (2021). mTOR and S6K1 mediate assembly of the translation preinitiation complex through dynamic protein interchange and ordered phosphorylation events. Cell.

[31] Avruch J, Belham C, Weng Q, Hara K, Yonezawa K (2001). The p70 S6 kinase integrates nutrient and growth signals to control translational capacity. Prog. Mol. Subcell Biol..

[32] Fingar DC, Salama S, Tsou C, Harlow E, Blenis J (2002). Mammalian cell size is controlled by mTOR and its downstream targets S6K1 and 4EBP1/eIF4E. Genes Dev..

[33] Porstmann T (2008). SREBP activity is regulated by mTORC1 and contributes to Akt-dependent cell growth. Cell Metab..

[34] Peterson TR (2011). mTOR complex 1 regulates lipin 1 localization to control the SREBP pathway. Cell.

[35] Zhang, Y. *et al*. Xyloketal B Attenuates Fatty Acid-Induced Lipid Accumulation via the SREBP-1c Pathway in NAFLD Models. *Mar. Drugs* **15**. 10.3390/md15060163. (2017). 10.3390/md15060163 PMC5484113 28587208

[36] Caron A, Richard D, Laplante M (2015). The Roles of mTOR Complexes in Lipid Metabolism. Annu. Rev. Nutr..

[37] Kim K, Pyo S, Um SH (2012). S6 kinase 2 deficiency enhances ketone body production and increases peroxisome proliferator-activated receptor alpha activity in the liver. Hepatology.

[38] Um SH (2004). Absence of S6K1 protects against age- and diet-induced obesity while enhancing insulin sensitivity. Nature.

[39] Hara K (1998). Amino acid sufficiency and mTOR regulate p70 S6 kinase and eIF-4E BP1 through a common effector mechanism. J. Biol. Chem..

[40] Kimball SR (2001). Regulation of translation initiation by amino acids in eukaryotic cells. Prog. Mol. Subcell Biol..

[41] Lynch CJ (2002). Leucine is a direct-acting nutrient signal that regulates protein synthesis in adipose tissue. Am. J. Physiol. Endocrinol. Metab..

[42] Zoncu R (2011). mTORC1 senses lysosomal amino acids through an inside-out mechanism that requires the vacuolar H(+)-ATPase. Science.

[43] Tsun ZY (2013). The folliculin tumor suppressor is a GAP for the RagC/D GTPases that signal amino acid levels to mTORC1. Mol. Cell.

[44] Gwinn DM (2008). AMPK phosphorylation of raptor mediates a metabolic checkpoint. Mol. Cell.

[45] Kimura N (2003). A possible linkage between AMP-activated protein kinase (AMPK) and mammalian target of rapamycin (mTOR) signalling pathway. Genes Cells.

[46] Long X, Lin Y, Ortiz-Vega S, Yonezawa K, Avruch J (2005). Rheb binds and regulates the mTOR kinase. Curr. Biol..

[47] Garratt M, Nakagawa S, Simons MJ (2016). Comparative idiosyncrasies in life extension by reduced mTOR signalling and its distinctiveness from dietary restriction. Aging Cell.

[48] Yun YS (2016). mTORC1 coordinates protein synthesis and immunoproteasome formation via PRAS40 to prevent accumulation of protein stress. Mol. Cell.

[49] Schmittgen TD, Livak KJ (2008). Analyzing real-time PCR data by the comparative C(T) method. Nat. Protoc..

[50] Kimball SR, Shantz LM, Horetsky RL, Jefferson LS (1999). Leucine regulates translation of specific mRNAs in L6 myoblasts through mTOR-mediated changes in availability of eIF4E and phosphorylation of ribosomal protein S6. J. Biol. Chem..

